# Bohmian Chaos and Entanglement in a Two-Qubit System

**DOI:** 10.3390/e27080832

**Published:** 2025-08-06

**Authors:** Athanasios C. Tzemos, George Contopoulos, Foivos Zanias

**Affiliations:** 1 Research Center for Astronomy and Applied Mathematics of the Academy of Athens, Soranou Efessiou 4, GR-11527 Athens, Greece; gcontop@academyofathens.gr (G.C.); foivos.zanias@student.uva.nl (F.Z.); 2 Institute of Physics, University of Amsterdam, Science Park 904, 1098 XH Amsterdam, The Netherlands

**Keywords:** chaos, Bohmian quantum mechanics, entanglement

## Abstract

We study in detail the critical points of Bohmian flow, both in the inertial frame of reference (Y-points) and in the frames centered at the moving nodal points of the guiding wavefunction (X-points), and analyze their role in the onset of chaos in a system of two entangled qubits. We find the distances between these critical points and a moving Bohmian particle at varying levels of entanglement, with particular emphasis on the times at which chaos arises. Then, we find why some trajectories are ordered, without any chaos. Finally, we examine numerically how the Lyapunov Characteristic Number (LCN) depends on the degree of quantum entanglement. Our results indicate that increasing entanglement reduces the convergence time of the finite-time LCN of the chaotic trajectories toward its final positive value.

## 1. Introduction

Bohmian Quantum Mechanics (BQM) is an alternative interpretation of quantum mechanics, which predicts deterministic trajectories for the quantum particles [1,2,3,4,5]. These trajectories are guided by the wavefunction Ψ, which describes a given quantum system, i.e., a solution of the Schrödinger equation, according to a set of first-order-in-time differential equations, the following Bohmian equations of motion:

(1) midxidt=ħIm∇iΨΨ,

where $x_{i}$ is the position of the *i*-th particle and $m_{i}$ its mass. BQM predicts the same experimental results as the standard quantum mechanics (SQM) and has attracted the interest of many authors, both from theoretical and practical standpoints [6,7,8].

Chaos in quantum mechanics has remained an open and actively investigated problem for several decades. In the standard (Copenhagen) formulation of quantum theory, the evolution of a system is governed by the unitary propagation of the wavefunction under the Schrödinger equation. However, this framework does not admit a notion of trajectory, making the study of dynamical complexity and chaotic behavior fundamentally different from the classical case. As a result, quantum chaos is typically characterized through indirect, statistical, or information-theoretic indicators [9,10,11]. Among the most prominent tools are spectral statistics, such as the distributions of the successive energy levels [12], the spread of a quantum state over a given basis (participation ratio) and the operator growth, especially in many-body systems, where out-of-time-order correlators (OTOCs) [13] are used to find how initially localized operators spread under time evolution. While these approaches yield valuable insights, they do not provide a unified or universally accepted definition of chaos, nor do they offer a trajectory-based picture analogous to classical chaotic motion.

In contrast, Bohmian Quantum Mechanics provides a deterministic and trajectory-based formulation of quantum theory, where particles follow well-defined paths guided by the quantum wavefunction. This framework introduces a classical-like phase space structure into quantum mechanics and allows one to explore quantum dynamics using the full arsenal of tools from nonlinear dynamical systems theory. Bohmian trajectories evolve under a set of first-order, generally non-autonomous and nonlinear differential equations, making them capable of exhibiting both regular and chaotic motion. As such, concepts like Lyapunov exponents, stable/unstable manifolds, etc., become directly applicable in the quantum regime. This makes BQM uniquely suited to studying the dynamical origins of quantum chaos in a physically transparent and geometrically clear manner. Thus, BQM offers new perspectives and tools for understanding complex quantum behavior in systems ranging from single-particle models to quantum many-body dynamics.

The origin of Bohmian chaos has been the subject of many works in the past [14,15,16,17,18,19,20] where it was noticed that chaos is produced due to the interaction of a Bohmian particle with the nodal points of the corresponding wavefunction (where $\Psi =\Psi_{R}+i\Psi_{I}=0$). However, as we showed in [21], it is not the nodal point *N* itself, but its accompanying unstable point in the frame of reference of the nodal point, the ‘X-point’, which is responsible for the generation of chaos. The points *N* and *X* form a ‘nodal point-X-point complex’ (NPXPC), a special dynamical structure of the Bohmian flow in the close neighborhood of *N*. Whenever a quantum particle comes close to an NPXPC, its trajectory deviates due to the interaction with the X-point. This interaction is accompanied by a shift of the ‘finite time Lyapunov characteristic number’ (χ). The cumulative effect of many such scattering events is the convergence of χ to the LCN at a positive value, which is the hallmark of chaos.

In our works, we studied different wavefunctions of the unperturbed 2-d quantum harmonic oscillator (which is the most well-studied system in the field of Bohmian chaos [22]) through the prism of the NPXPC mechanism. We note that while this system is classically analytically solvable, i.e., it has no chaos, its Bohmian counterpart has a very rich nonlinear dynamics with both ordered and chaotic trajectories. Most results were found in the case of a wavefunction with a single nodal point (see Section 2), since in this case we know analytically the position of the nodal point in space for every time. We also studied more complicated wavefunctions with two, three, and multiple nodal points scattered around the phase space with different geometries [23]. In this case, the position of the nodal points is found, in general, in a numerical way. However, we found a special wavefunction composed of coherent states of the harmonic oscillator, where again we know the position of the nodal points analytically [24,25]. With a proper choice of physical parameters, this wavefunction resembles a system of two entangled qubits, something very important for applications in quantum information theory [26].

Furthermore, we found that in the above case, all chaotic trajectories are ergodic. Namely, any two different chaotic trajectories acquire practically the same distribution of points in the long run, something that we showed in [25,27] by using their ‘colorplots’, where different colors represent the number of trajectory points in a bin of a dense square grid. The distance between the produced colorplots was measured by using the Frobenius distance [28] between the corresponding underlying matrices. Thus, chaotic trajectories give the same colorplot. On the other hand, the ordered trajectories have the shape of deformed Lissajous figures and give different colorplots, i.e., they are not ergodic. We note here that in the classical case, ergodicity [29] is defined in autonomous systems with a finite phase space. In BQM, the available phase space has, strictly speaking, infinite size but in practice is confined in the “ effective support” of the wavefunction, i.e., the region where the probability density of the wavefunction is not extremely small (see also [30] for ergodicity in BQM).

In all the above cases, we studied in detail the NPXPC mechanism and its effectiveness in understanding the generation of Bohmian chaos. The main result is that in general, chaos emerges from the action of the X-points on the Bohmian trajectories, and the NPXPC mechanism accounts for the general profile of the LCN. However, there were some minor events in the finite time LCN, in which the X-point was far from the trajectory. These events could not be understood until our work [31] where we showed that besides the X-points, there are also unstable fixed points in the inertial frame of reference. We called them ‘Y-points’ and found that they are responsible for these minor contributions to the LCN. The combined action of *X*- and Y-points provides a full explanation of the profile of the LCN. On the other hand, if the trajectory of a particle never approaches an X-point or a Y-point, then the trajectory is ordered and not chaotic.

Both in the case of a single-node wavefunction (there is only one Y-point whose position is found analytically) and in various multinodal wavefunctions, we found that, in general, the Y-points are distant from the X-points. But the generality of the above result remains an open problem. As we will show in the present paper, there are cases where the contribution of the Y-points in chaos production is comparable to that of the X-points.

In particular, we will study the case of infinitely many N,X and Y-points of two entangled qubit states. We will provide the analytical formulae for their positions, in addition to those of the nodal points. As we will show, in this case, all the critical points are close to each other at any time *t*. Moreover, every nodal point has two X-points, one ahead of it and one behind it. All these new findings make the study of the onset of chaos much more complicated. Thus, in order to understand it better, we will show in detail some representative cases of approaches to the nodal points, X-points and Y-points.

Entanglement is a fundamental aspect of quantum mechanics, endowing quantum systems with unique properties for information storage, processing, and transmission [32]. It is of immense theoretical and technological interest, as it underlies many key developments in quantum computing and communication [33,34,35,36]. In the context of Bohmian mechanics [7,37,38,39,40], entanglement is essential for inducing complex, potentially chaotic behavior in the trajectories. Without entanglement, the system of Bohmian equations becomes decoupled, and particle dynamics reduce to independent, regular motion. Unlike classical systems, where chaos can emerge from nonlinear interactions between degrees of freedom, entanglement has no classical counterpart. Therefore, studying how entanglement affects chaos in Bohmian trajectories provides a unique window into the quantum origins of dynamical complexity. We are going to show some results on the relation between entanglement and the behavior of the LCN for various sets of initial conditions, something that is an open problem in BQM.

The structure of this paper is the following: In Section 2, we provide a short review of the NPXPC mechanism, including the Y-points, in the case of a single nodal point, and then pass to the case of two entangled qubits, where we provide the formulae for the positions of the Y-points. Then, we study some characteristic trajectories for very small and intermediate values of entanglement and give their distances from N,X,Y at different scattering events as a function of time, discussing their effects on the shape of the trajectories and on the finite time LCN. At the end of Section 2, we discuss the ergodic character of the chaotic Bohmian trajectories. In Section 3, we discuss the ordered trajectories and their different origin depending on the commensurability of the frequencies of the oscillator. In Section 4, we provide numerical evidence that quantum entanglement does not affect the value of the LCN itself in a certain way but affects the time of its convergence to a final value. In Section 5, we summarize our results and draw our conclusions. Finally, in Appendix A, we give details about the detection of the X-points, and in Appendix B, we discuss the periodicity of the trajectories in the case of commensurable frequencies.

## 2. The Mechanism of Chaos in BQM

As we mentioned in the introduction, the NPXPC is a geometrical structure that characterizes the local geometry of the Bohmian flow near a moving node. The NPXPC changes in time due to the non-autonomous nature of the Bohmian equations of motion. In fact, the distance between *N* and *X* decreases as the velocity of the nodal point increases, while the nature of the nodal point alternates between repeller and attractor [21].

**Figure 1:**
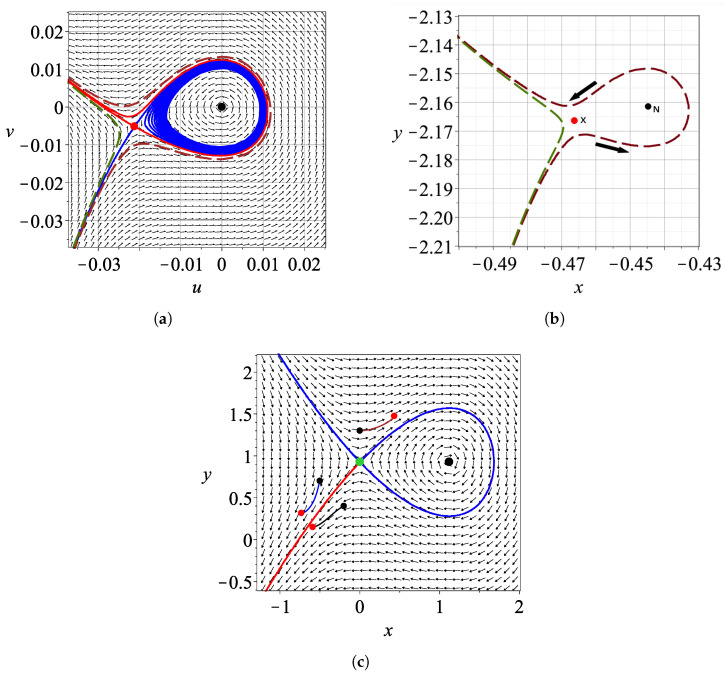
(**a**) The Bohmian flow and the asymptotic curves of the X-point (red unstable, and blue stable) corresponding to the wavefunction $\Psi =a\Psi_{00}+b\Psi_{10}+c\Psi_{11}$ with $a=b=1,c=\sqrt{2}/2$ at t=1.5, in the reference frame of the nodal point ($u=x-x_{N}$, $v=y-y_{N}$). The black dot at u=v=0 is the nodal point, while the red dot at u=−0.2123,v=−0.5132 is the X-point. We also plot two trajectories approaching the X-point and getting deflected by it (dark green and burgundy dashed curves). (**b**) The same trajectories in the inertial frame of reference and the *X* and *N* points at t=1.5. (**c**) The corresponding Y-point and its stable (blue) and unstable (red) manifolds in the inertial frame of reference at t=1.5 together with arcs of 3 trajectories close to the Y-point (starting at the small red dots and ending at the small black dots). We note that both in (**a**,**c**), the blue and red asymptotic curves may come arbitrarily close to each other, but they never overlap, as ensured by the uniqueness theorem of differential equations.

An example of such an NPXPC is presented in Figure 1, where we examine the case of a two-dimensional quantum harmonic oscillator corresponding to the classical Hamiltonian

(2) $$H=\frac{1}{2}\left({\dot{x}}^{2}+\omega_{x}^{2}x^{2}+{\dot{y}}^{2}+\omega_{y}^{2}y^{2}\right)$$

with a wavefunction of the form

(3) $$\Psi =a\Psi_{0,0}+b\Psi_{1,0}+c\Psi_{1,1},$$

where $\Psi_{m,n}\left(x,y\right)=\Psi_{m}\left(x\right)\Psi_{n}\left(y\right)$ and $\Psi_{m}\left(x\right),\Psi_{n}\left(y\right)$ are the 1-d energy eigenstates of the oscillator in *x* and *y* coordinates, respectively, i.e.,

(4) $$\Psi_{m,n}=\prod_{q=x}^{y}N_{q}\exp\left(-\frac{\omega_{q}q^{2}}{2\hbar }\right)\exp\left(-\frac{i}{\hbar }E_{r}t\right)H_{r}\left(\sqrt{\frac{M_{q}\omega_{q}}{\hbar }}q\right),$$

and r=m,n (integers) for *x* and *y*, respectively, and the normalization constant $N_{q}=\frac{{\left(M_{q}\omega_{q}\right)}^{\frac{1}{4}}}{\pi \hbar \sqrt{2^{r}r!}}$. $H_{m},H_{n}$ denote the Hermite polynomials in $\sqrt{\frac{M_{x}\omega_{x}}{\hbar }}x$ and $\sqrt{\frac{M_{y}\omega_{y}}{\hbar }}y$ of degrees *m* and *n*, respectively. Finally, the energy of $\Psi_{m,n}$ is $E_{m,n}=E_{m}+E_{n}=\left(\frac{1}{2}+m\right)\hbar \omega_{x}+\left(\frac{1}{2}+n\right)\hbar \omega_{y}.$

The wavefunction (3) has been extensively studied in the field of Bohmian chaos since it has only one nodal point whose coordinates are given analytically [22,41]:

(5) $$x_{N}=-\frac{a\sqrt{2}\sin\left[\left(\omega_{x}+\omega_{y}\right)t\right]}{2\sqrt{\omega_{x}}\;b\sin\left[\omega_{y}t\right]},\;y_{N}=-\frac{b\sqrt{2}\sin\left[\omega_{x}t\right]}{2\sqrt{\omega_{y}}\;c\sin\left[\left(\omega_{x}+\omega_{y}\right)t\right]},$$

simplifying significantly the study of chaos generation with the NPXPC mechanism.

To find the location of the X-point at a fixed time *t*, we begin with the original Bohmian velocity field defined by the system $\frac{dx}{dt}$, $\frac{dy}{dt}$, and pass to a co-moving reference frame centered at the nodal point by defining new variables $u=x-x_{\mathrm{nod}}$ and $v=y-y_{\mathrm{nod}}$, and transforming the equations to

(6) $$\frac{du}{dt}=\frac{dx}{dt}-\frac{dx_{nod}}{dt},\;\frac{dv}{dt}=\frac{dy}{dt}-\frac{dy_{nod}}{dt},$$

where $\frac{dx_{nod}}{dt}$ and $\frac{dy_{nod}}{dt}$ are the velocities of the nodal point in the inertial frame of reference. The X-point is then defined as the fixed point of the flow in this moving frame and is determined numerically by solving the equations

(7) $$\frac{du}{dt}=0,\;\frac{dv}{dt}=0.$$

Once the X-point is found, we calculate the Jacobian matrix of the Bohmian flow in the (u,v) coordinates, evaluated at its location. The eigenvalues and eigenvectors of this matrix define the local linear dynamics and provide the stable and unstable eigendirections. To visualize the corresponding asymptotic curves, we integrate the equations of motion by using a fictitious time parameter *s*, associated with an autonomous system obtained by freezing the time dependence of the Bohmian flow at the fixed time *t* (Figure 1a). This approach, known as the ‘adiabatic approximation’, assumes that the time variation of the flow is sufficiently slow in the neighborhood of *t*, allowing us to study the local scattering of Bohmian trajectories by the NPXPC as if the structure were momentarily frozen. The trajectories starting close to the lower stable asymptotic curve approach the X-point and deviate to the left or to the right close to the unstable asymptotic curves of *X* (Figure 1a) in the frame of reference (u,v) centered at the nodal point *N*. In the inertial frame of reference (x,y), these orbits are shown in Figure 1b, while *N* and *X* have particular positions at time *t*.

In this case, we see that the nodal point acts as a repeller. The unstable asymptotic curves of the X-point, which govern the divergence of the trajectories in forward time and are shown in red, while the stable ones, which attract trajectories toward the X-point, are shown in blue. Some trajectories become temporarily trapped near the nodal point and exhibit spiral motion around it as the nodal point moves, a phenomenon known as a ‘Bohmian vortex’. This vortex behavior persists while the trajectories remain within the region bounded by the nodal point and the X-point. However, the trajectories exit from this region as the nodal point accelerates and tends to infinity (when the denominator of $x_{N}$ or $y_{N}$ becomes zero).

With the NPXPC mechanism, we were able to study a variety of cases and explain in general the form of the time evolution of the finite-time Lyapunov characteristic number. However, in [31] we showed the existence of a fixed point of the Bohmian flow in the inertial frame of reference, which is also unstable. This point, referred to as the ‘Y-point’, is defined as the solution of the system

(8) $$\frac{dx}{dt}=\frac{dy}{dt}=0.$$

In the case of the wavefunction with a single node (3), the position of the Y-point is found analytically:

(9) $$x_{Y}=0,\;y_{Y}=-\frac{b\sqrt{2}\sin\left(\omega_{x}t\right)}{2c\sqrt{\omega_{y}}\sin\left[\left(\omega_{x}+\omega_{y}\right)t\right]}.$$

We note that in this case the Y-point has the same values of *y* as the nodal point, i.e., $y_{Y}=y_{N}$. In this frame of reference (*x*, *y*) the asymptotic curves of the Y-point are shown in Figure 1c for a fixed time *t*. We observe that they come so close that they appear to overlap, which would violate the uniqueness theorem for solutions; however, they never actually intersect.

In the above case, the node *N* and its accompanying X-point are, in general, far from the Y-point. Thus, the latter was found to play a secondary role in the generation of chaos [31]. Similar results were found in other wavefunctions of the 2-d quantum harmonic oscillator (2) with two or more nodal points randomly located on the x−y plane.

Another important case that we studied in the quantum analogue of a classical 2-d harmonic oscillator is that of entangled qubits made by coherent states along the *x* and *y* axes.

We remind the reader that a one-dimensional coherent state in *x* direction is a special quantum state of the quantum harmonic oscillator, characterized by minimum uncertainty. As such, its evolution closely resembles that of a classical harmonic oscillator. Technically, a coherent state |α(t)〉 is defined as the eigenstate of the annihilation operator $\hat{\alpha }$, associated with the (generally complex) eigenvalue α(t):

(10) $$\hat{\alpha }|\alpha \left(t\right)\rangle =\alpha \left(t\right)|\alpha \left(t\right)\rangle ,$$

where $\alpha \left(t\right)=\alpha_{0}e^{-i\omega t}$ is a complex eigenvalue, since $\hat{\alpha }$ is non-Hermitian [42].

When expanded in terms of the energy eigenstates, the corresponding wavefunction is

(11) $$Y\left(x,t\right)=e^{-{\left|\alpha \right|}^{2}/2}\sum_{n=0}^{\infty }\frac{\alpha {\left(t\right)}^{n}}{\sqrt{n!}}\psi_{n}\left(x\right),$$

where $\psi_{n}\left(x\right)$ are the usual energy eigenfunctions of the quantum harmonic oscillator with the corresponding Hermite polynomials [43]

(12) $$\psi_{n}\left(x\right)={\left(\frac{1}{2^{n}n!}\right)}^{1/2}{\left(\frac{M_{x}\omega_{x}}{\pi \hbar }\right)}^{1/4}e^{-\frac{M_{x}\omega_{x}^{2}}{2\hbar }}H_{n}\left(\sqrt{\frac{M_{x}\omega_{x}}{\hbar }}x\right).$$

This expansion reflects the fact that coherent states are superpositions of all number states, weighted by a Poissonian distribution centered around ${\left|\alpha \right|}^{2}$.

By using the properties of the Hermite polynomials and summing over *n* in (12), one obtains the wavefunction in the position representation:

(13) Y(x,t)=mωπħ14exp−mω2ħx−2ħmωRe[α(t)]2+i2mωħIm[α(t)]x+ζ(t),

where

(14) Re[α(t)]=a0cos(σ−ωt),Im[α(t)]=a0sin(σ−ωt),ζ(t)=12a02sin2(ωt−σ)−ωt.

We now define the following position wavefunctions:

(15) $$\begin{array}{c} Y_{R}=Y_{R}\left(i,t\right)\equiv Y\left(i,t;\omega =\omega_{i},m=m_{i},\sigma =\sigma_{i}\right),\;i=x,y,\\ Y_{L}=Y_{L}\left(i,t\right)\equiv Y\left(i,t;\omega =\omega_{i},m=m_{i},\sigma =\sigma_{i}+\pi \right),\;i=x,y, \end{array}$$

which describe one-dimensional coherent states that begin their motion at the right or left extreme point of the classical oscillation, along the *x* or *y* directions, respectively. With these, we construct a two-dimensional entangled wavefunction:

(16) $$\Psi =c_{1}Y_{R}\left(x,t\right)Y_{L}\left(y,t\right)+c_{2}Y_{L}\left(x,t\right)Y_{R}\left(y,t\right),$$

which displays quantum entanglement between the *x* and *y* degrees of freedom (for systems with similar wavefunction see [44]. However, since the inner product between two arbitrary coherent states is generally non-zero, care must be taken when interpreting this as a qubit system. In the case of a common amplitude $a_{0}$ along both directions, and with $\sigma_{x}=\sigma_{y}=0$, the inner product between $Y_{R}$ and $Y_{L}$ becomes $\langle Y_{R}|Y_{L}\rangle =\exp\left(-2a_{0}^{2}\right)$. Thus, for $a_{0}=5/2$, the overlap becomes negligibly small (of order $10^{-6}$), making the two states practically orthogonal. Consequently, the wavefunction (16) can be seen as a superposition of two nearly orthogonal product states, thereby effectively realizing a system of two entangled qubits (for further details, see [24,39]). From now on we work with m=ħ=1.

The above system, besides its technological applications, mainly in quantum optics [42] is very useful for the study of Bohmian chaos since:

1. It has infinitely many nodal points. This is due to the infinitely many energy eigenstates inside the coherent states (11). The positions of the nodal points are given analytically
  (17) $$x_{nod}=\frac{\sqrt{2}\left(k\pi \;\cos\left(\omega_{y}\;t\right)+\sin\left(\omega_{y}\;t\right)\ln\left(\left|\frac{c_{1}}{c_{2}}\right|\right)\right)}{4\sqrt{\omega_{x}}a_{0}\;\sin\left(\omega_{xy}t\right)},$$
  (18) $$y_{nod}=\frac{\sqrt{2}\left(k\pi \;\cos\left(\omega_{x}t\right)+\sin\left(\omega_{x}t\right)\ln\left(\left|\frac{c_{1}}{c_{2}}\right|\right)\right)}{4\sqrt{\omega_{y}}a_{0}\;\sin\left(\omega_{xy}\;t\right)},$$
  with k∈Z, *k* even for $c_{1}\cdot c_{2}<0$ or odd for $c_{1}\cdot c_{2}>0$ and $\omega_{xy}\equiv \omega_{x}-\omega_{y}$.
2. As shown in [24], this model has very rich Bohmian dynamics. In the case of commensurable frequencies, all trajectories are periodic, while for non-commensurable frequencies, we observe the coexistence of order and chaos. From now on, we choose to work with positive $c_{1},c_{2}$, $\omega_{x}=1$, and $\omega_{y}=\sqrt{3}$ (i.e., with irrational frequencies). Therefore, k=...,−5,−3,−1,1,3,5,....
3. The amount of entanglement in this system can be analytically calculated [24]. Entanglement is maximized when $c_{1}=c_{2}=\sqrt{2}/2$, while entanglement is zero for $c_{1}\cdot c_{2}=0$.

A new result of the present work is that if we calculate the fixed points of the Bohmian flow in the inertial frame of reference, we find that there are also infinitely many Y-points between the successive nodal points, whose positions are given analytically by the following equations:

(19) $$x_{Y}=\frac{\sqrt{2}\left(2k^{\prime }\pi \;\cos\left(\omega_{y}\;t\right)+\sin\left(\omega_{y}\;t\right)\ln\left(\left|\frac{c_{1}}{c_{2}}\right|\right)\right)}{4\sqrt{\omega_{x}}a_{0}\;\sin\left(\omega_{xy}t\right)},$$

(20) $$y_{Y}=\frac{\sqrt{2}\left(2k^{\prime }\pi \;\cos\left(\omega_{x}t\right)+\sin\left(\omega_{x}t\right)\ln\left(\left|\frac{c_{1}}{c_{2}}\right|\right)\right)}{4\sqrt{\omega_{y}}a_{0}\;\sin\left(\omega_{xy}\;t\right)},$$

with $2k^{\prime }=\ldots -4,-2,0,2,4\ldots$

**Figure 2:**
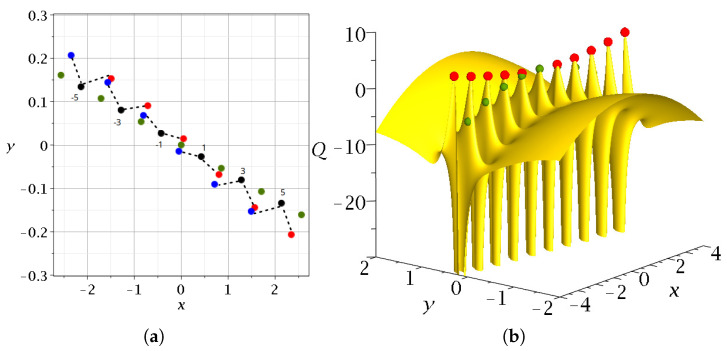
(**a**) The critical points *N* (black), *X* (blue), and *Y* (green) of the Bohmian flow on the x−y plane at t=1.5 for $c_{2}=\sqrt{2}/2$ and $\omega_{x}=1,\omega_{y}=\sqrt{3}$. (**b**) The corresponding 3d surface of the quantum potential *Q* in the region of a line containing nodal points. The nodal points are at $Q_{N}=-\infty$. The red points are the X-points, while the green points are the Y-points. We observe that every X-point is practically on the top of the local maximum close to a node [45]. The origin (0,0) corresponds to a Y-point.

Therefore, the Y-points are in the middle of two successive nodal points. This is seen in Figure 2, where we give the positions of the nodal points, the X-points and the Y-points at a particular time t=1.5. Near every nodal point *N*, there are two X-points, one on the right (red) and one on the left (blue). These points are close to the Y-points between two successive nodal points (Figure 2a). In Appendix A, we describe how the X-points are calculated. In Figure 2a, we see that the red X-point (on the right of a nodal point *N*) is very close to the blue X-point of the previous nodal point.

The positions of the *N*, *X* (red) and Y-points (green) on the quantum potential surface *Q* [45] (*Q* is defined as $Q=-\frac{\hbar^{2}}{2m}\frac{\nabla^{2}\left|\Psi \right|}{\left|\Psi \right|}$, where *ℏ* is Plancks constant, *m* is the mass and Ψ is the wavefunction [46,47,48,49]. We work with m=ħ=1.) are shown in Figure 2b. At the origin (x=0,y=0) we have a Y-point with $k^{\prime }=0$. As |k| and $|k^{\prime }|$ increase, the *Q* values of the red points increase, and those of the Y-points decrease. Further away from the lines of the nodal points, the X-points and the Y-points, *Q* increases smoothly. We observe that beside the central Y-point lying at the origin of the x−y plane, the X-points are located at higher values of *Q* than the Y-points. This shows that in this model, we have two kinds of unstable points that produce chaos. However, even in this case, the total force F=−∇(V+Q) acting on a Bohmian particle is larger at the X-points than at the Y-points.

The case of entangled qubits has a new interesting feature regarding Bohmian chaos: its Y-points are always close to the X-points, something that was not the case in the wavefunction of a single nodal point. This means that both the X-points and the Y-points contribute practically equally to producing chaos.

**Figure 3:**
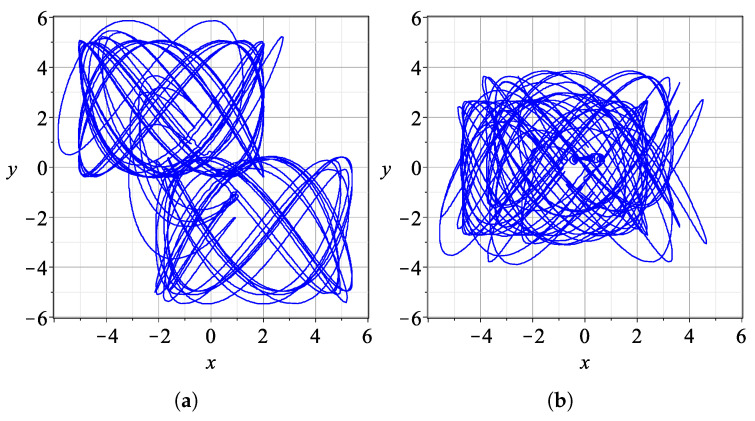
Trajectories of particles in the inertial frame x,y for a time interval t∈[0,200]. (**a**) For $c_{2}=0.001$ with x(0)=0,y(0)=3. (**b**) For $c_{2}=0.3$ with x(0)=−2.5654,y(0)=3.6585.

In Figure 3, we show two characteristic trajectories for t∈[0,200] for different degrees of the entanglement parameter (a) with $c_{2}=0.001$ (weak entanglement) and (b) $c_{2}=0.3$ (moderate entanglement).

If there is no entanglement ($c_{2}=0$), the trajectories in the x−y plane are Lissajous figures (all critical points are at *∞*). If the entanglement is small, the trajectories form Lissajous-like figures for some intervals of time. When the particle is far from all nodal points and from the *X* and *Y* points, the trajectory is very close to a Lissajous figure, but when it approaches the *N*, *X* and Y-points, the trajectory undergoes a change. Some close approaches lead to very different Lissajous-like forms (Figure 3a,b). But when $c_{2}$ is close to its maximum ($c_{2}=\sqrt{2}/2$), the trajectories have no time to form any Lissajous-like forms.

### 2.1. A Typical Bohmian Chaotic Trajectory

**Figure 4:**
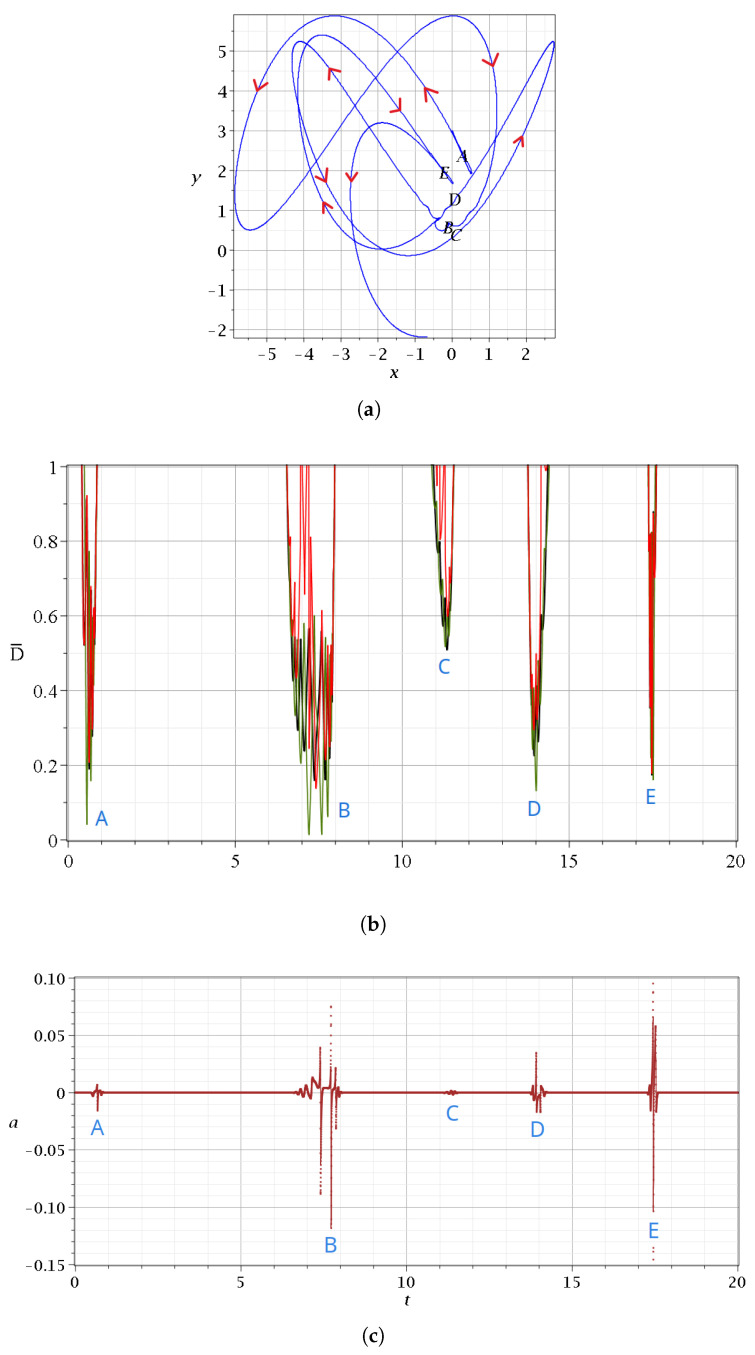
(**a**) A Bohmian trajectory of a particle starting with $c_{2}=0.001,\omega_{x}=1,\omega_{y}=\sqrt{3}$ at x(0)=0,y(0)=3 up to t=20, following the directions of the red arrows. (**b**) The distance between the particle and the closest nodal point (black), the closest X-point (red), and the closest Y-point (green) for the same time interval. (**c**) The variations of the stretching number *a*. We observe that its spikes take place when the distances between the critical points and the particle are minimized. These happen at the points *A*–*E* shown on the trajectory.

In Figure 4, we give the details of some approaches of a Bohmian particle to N,X,Y in the case $c_{2}=0.001$ for a time interval t∈[0,20]. The trajectory is given in Figure 4a, and in Figure 4b, we give the distances from the closest nodal point (black curve), the closest X-point (red curve), and the closest Y-point (green curve) at every time. The approaches take place near the points *A*, *B*, *C*, *D*, and *E* of the trajectory (Figure 4a). We note that near the time of an approach to a particular nodal point *N*, we have also approaches to some nearby *N*, *X*, and *Y* points. We call such a set of approaches an ‘event’. The main events are *A*, *B*, *D*, and *E* at minimum distances of the order $\tilde{D}=0.2$, while during the event *C*, the minimum distance is close to $\tilde{D}=0.6$.

In Figure 4c, we give the corresponding variations of the stretching number, which lead to chaos. We remind the reader that the stretching number is related to the Lyapunov characteristic number: in particular, if we take two nearby trajectories at $t=t_{0}$ and their deviation vector $\xi_{k}$ at the times t=sΔt,s=1,2,… then we define the ‘finite time Lyapunov characteristic number’ χ [21] as

(21) $$\chi =\frac{1}{t}\sum_{i=1}^{s}a_{i},$$

where

(22) $$a_{s}=\ln\left|\frac{\xi_{s+1}}{\xi_{s}}\right|$$

is the ‘stretching number’. Thus, a/Δt is a ‘one-step Lyapunov characteristic number’. The LCN itself is the limit of χ at s→∞ and is zero for ordered trajectories, while it is positive for chaotic trajectories (we work with Δt=0.01).

**Figure 5:**
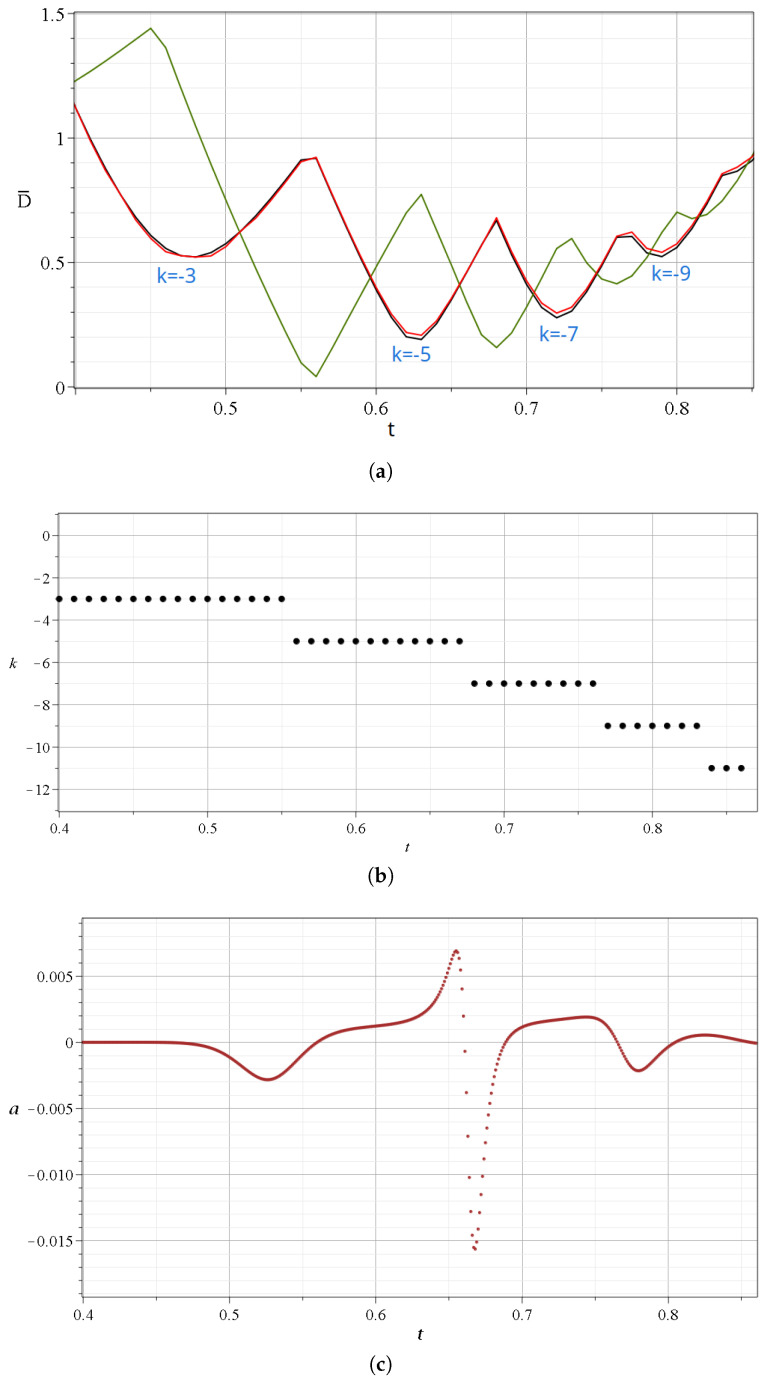
(**a**) Zoom in of Figure 4a at the scattering event *A* taking place at t∈[0.4,0.8]. (**b**) The indices *k* of the closest nodal point and (**c**) the values of the stretching number *a* during this time interval.

We note that the closest points *N*, *X*, and *Y* to the trajectory of the particle change over time during a single scattering event. This is shown in Figure 5a,b, where we see a zoom in the first event (A(t∈ [0.4–0.85])). During *A*, the involved nodal points are k=−3,−5,−7, and k=−9.

The details of Figure 5a are the following: The particle approaches first (at t=0.48) the nodal point k=−3 (Equations (17) and ()), and beyond that time, the distance from this nodal point increases. Then, at t=0.56, the moving particle comes close to the Y-point (minimum of green curve), with $2k^{\prime }=-4$, between the nodal points k=−3 and k=−5. After that time, the particle comes closer to the nodal point k=−5. Near the time of the minimum distance from the Y-point, we have the crossing of the two black curves k=−3 and k=−5 and of the corresponding X-curves (red). Then, at t=0.62, the distance from the point k=−5 is minimum. At t=0.67, we have a minimum of the green curve ($2k^{\prime }=-6$) and a corresponding crossing of the distances from the nodal points $\bar{D}$ (black curves) k=−5 and k=−7, and the corresponding X-points. Then, we have a minimum of the curve k=−7, another minimum of the green curve, and an approach to the nodal point k=−9. Beyond that time, all the distances from N,X,Y become large as prior to the event *A* (t<0.4) The closest nodal point for various times is given in Figure 5b. Therefore, during *A*, we have approaches to four nodal points and to three Y-points between them. Similar effects appear if we zoom in, the other events B,C,D,E.

During an event, we have a number of spikes consisting of increases and decreases in the stretching number *a* (Figure 4c). The variations of *a* during the event *A* are given in a zoom (Figure 5c). The value of *a* is negative and positive during the event, while it is practically zero before and after the event. The total contribution of the values of *a* to the LCN during this event is slightly negative. However, other events make a positive contribution.

**Figure 6:**
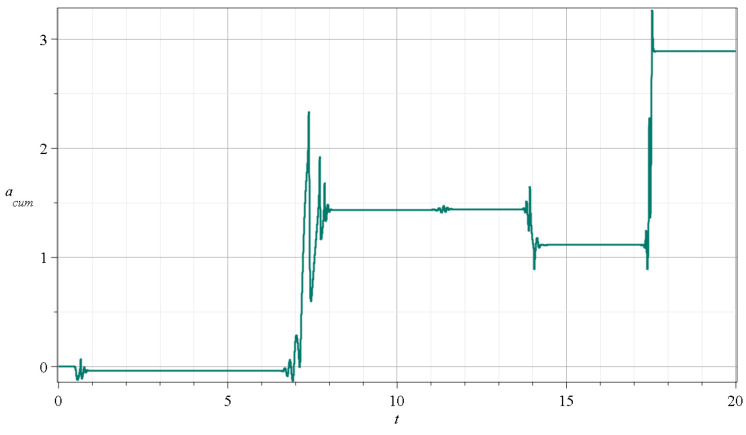
The cumulative stretching number $a_{cum}=\sum a$ in the case $c_{2}=0.001$ and for x(0)=0,y(0)=3 up to 20 time units.

In Figure 6, we give the sum of $a^{\prime }s$ (i.e., the $a_{cum}=\sum a$) (Equation (21)) for times up to t=20 ($a_{cum}$ shows more clearly the net effect of each scattering event on the production of chaos than the stretching number itself). We see that at every event, we have a decrease or an increase in this sum at the successive events, while between any successive events, the sum does not change. In fact, we have decreases at the events A,D, increases at the events B,E, and practically no change at the effect *C*. The value of the finite time LCN, χ, at any time *t* is the sum divided by the corresponding time (Equation (21)).

Therefore, $a_{cum}$ increases on average. The value of χ has some fluctuations, but it stabilizes at a constant value, which is the LCN>0 for chaotic trajectories. Therefore, chaos is a property that is established after several events and not by a single event (the case of ordered trajectories where LCN=0 is discussed in Section 3).

During the event *A*, the nodal points are moving and the positions of the nodal points are given at every Δt=0.01. At t=0, all the nodal points are at infinity. As *t* increases from t=0, they come to the central region (around x=y=0) for an interval of time (including the duration of the event *A* from t=0.4 to about t=0.82) and later at $t=t_{\infty }=\frac{\pi }{\omega_{2}-\omega_{1}}=\frac{\pi }{\sqrt{3}-1}\approx 4.3$, they escape again to infinity. Escapes happen at every multiple of $t_{\infty }$ and between two successive escapes we have, in general, an “event” (a set of close approaches to the *N*, *X*, and *Y* points within a small interval of time).

**Figure 7:**
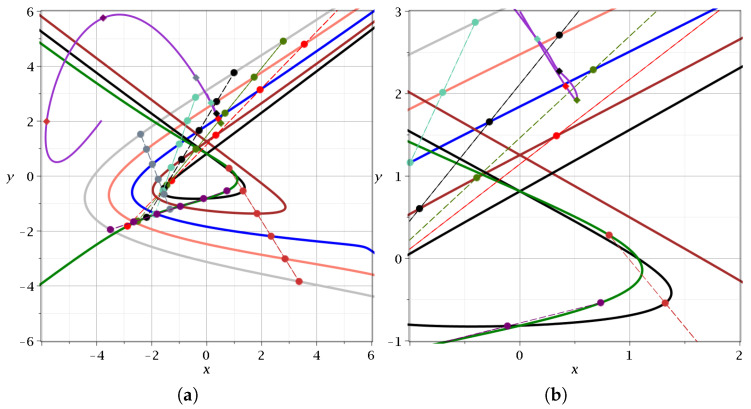
(**a**) The trajectories of the nodal points in the case $c_{2}=0.001,\omega_{x}=1,\omega_{y}=\sqrt{3}$ with k=1 (dark green), k=−1 (black), k=−3 (burgundy), k=−5 (blue), k=−7 (pink), and k=−9 (gray). The nodal points are given at the times t=0.4 (red, t=0.5 (green), t=0.6 (black), t=0.8 (cyan), t=1 (gray), t=2 (dark purple), and t=3 (dark orange). We also show a trajectory starting at x(0)=0,y(0)=3 for t∈[0,4.3]. (**b**) A zoom in showing the region where this trajectory comes close to a nodal point at t≃0.6 and forms a loop.

In Figure 7a we give the trajectories of the nodal points for k=1,−1,−3,−5,−7 and −9. At every time *t*, the nodes are along a straight line. We marked the lines at t=0.5 (red), t=0.6 (dark green), t=0.7 (black), t=0.8 (green), t=1 (gray), t=2 (yellow), and t=3 (red). Initially, the line of nodes makes an angle of about 37^o^ with the *x*-axis. In fact, as *t* tends to zero, the ratio $y_{N}/x_{N}$ tends to $\sqrt{\omega_{x}/\omega_{y}}=0.76=\tan37{}^{\circ}$, while *x* and *y* tend to +∞ for k<0, and to −∞ for k>0. If *t* tends to $t_{\infty }$, the ratio $y_{N}/x_{N}$ for large |k| tends to $\sqrt{\frac{\omega_{x}}{\omega_{y}}}\frac{\cos\left(\omega_{x}t_{\infty }\right)}{\cos\left(\omega_{y}t_{\infty }\right)}=-0.076=\tan\left(180{}^{\circ}-37{}^{\circ}\right)=\tan143{}^{\circ}$. The angle of the nodal line with the x-axis increases as *t* increases from t=0, and beyond t=1, it becomes larger than 180^o^, and approaches 180°+143° as $t\to t_{\infty }$. It is of interest to note that the trajectories of the nodal points with k>0 start at x=y=−∞ and those with k<0 start at x=y=+∞.

The trajectory of a particle starting at (x=0, y=3) forms a loop during event A, and then the particle moves up and to the left (we stop the calculations in this figure when $t=t_{\infty }$). The details are shown in Figure 7b. The moving particle is at the red point of its trajectory at t=0.5, and at the lowest point of the loop for t=0.6, when it is next to the nodal point k=−5 (compare with Figure 5a,b). Later, it moves upwards.

### 2.2. A Bohmian Chaotic Trajectory with a Vortex

Previously, we saw chaos generation in a typical Bohmian trajectory. Now, we study a trajectory containing a Bohmian vortex, a special phenomenon in Bohmian chaotic dynamics occurring whenever a trajectory comes very close to a moving nodal point and follows its motion forming a spiral around it for a given time interval.

**Figure 8:**
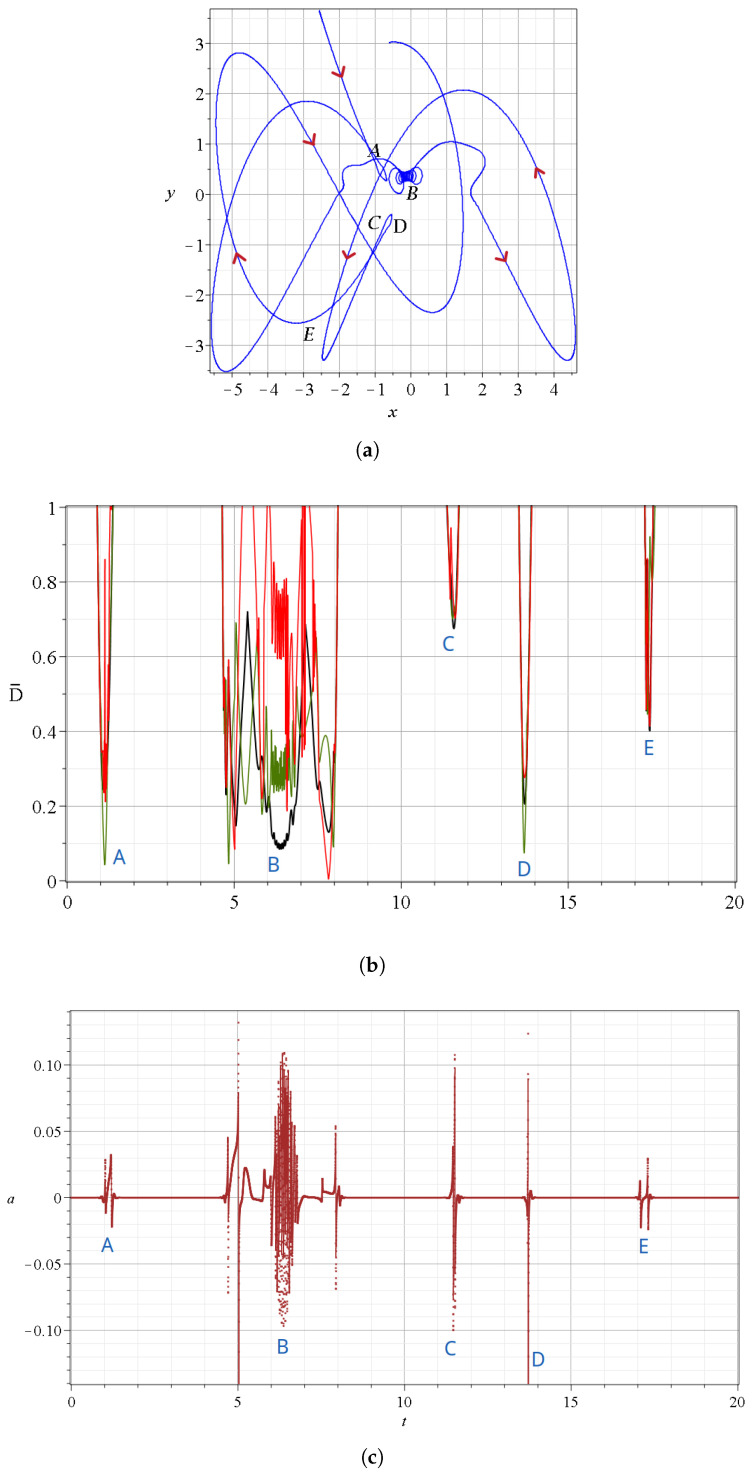
(**a**) A Bohmian trajectory starting at x(0)=−2.5654,y(0)=3.6585 up to t=20 for $c_{2}=0.3,\omega_{x}=1,\omega_{y}=\sqrt{3}$, following the directions of the red arrows. (**b**) The distance between the trajectory and the closest nodal point (black), the closest X-point (red), and the closest Y-point (green) for the same time interval. (**c**) The variations of the stretching number *a*.

In particular, in Figure 8, we show a set of events in the case $c_{2}=0.3$ for t∈[0,20]. Again, we have five events (*A*–*E*), as in our previous example. But here the event *B*, which corresponds to the Bohmian vortex, lasts a longer time (Figure 8b,c) than in the previous case with $c_{2}=0.001$.

**Figure 9:**
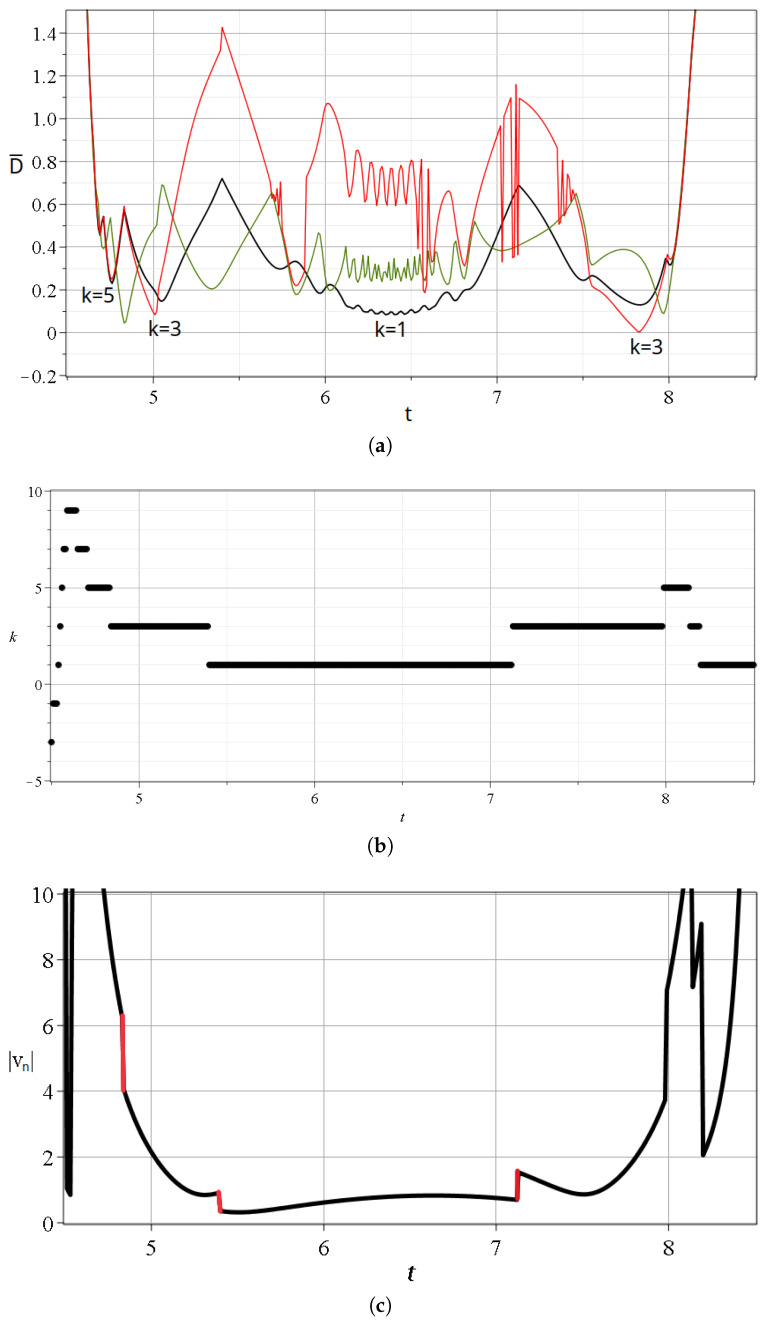
(**a**) Zoom of the event *B* that includes the Bohmian vortex taking place at t∈[6,7] (same colors as in Figure 8a). (**b**) the indices *k* of the closest nodal point and (**c**) the velocities of the closest nodal point during this time interval. Their abrupt changes are shown in red color.

More details are given in Figure 9a, where we see that the vortex exists for t∈[6,6.8]. During that time, the particle is closer to the nodal point (k=1) than to the corresponding X and Y-points. Before the spiral part of the trajectory, we had approaches to the nodal points k=5 and k=3 and to the corresponding *X* and *Y* points (Figure 9a,b). After the vortex, we have an approach to the nodal point k=3. There are further minima of distances to other nodal points before t=4.8 and after t=8.1, but these are at large distances $\tilde{D}$.

The velocities of the nearest nodal point *N* during the event *B* are given in Figure 9c. In general, the velocities are relatively large. However, during the approach to the nodal point k=1 (where we have the Bohmian vortex), they are quite small. Moreover, $\bar{D}$ shows an oscillatory behavior around the nodal point (black curve in Figure 8b), in contrast to the previous example, and because of that, the trajectory undergoes many spikes of the stretching number *a* during this period (Figure 8c). In Figure 9c, we see three abrupt changes in the velocity at the transitions from the node k=5 to k=3 (at t=4.9) from k=3 to k=1 (at t=5.4) and from k=1 to k=3 (at t=7.1) marked with red segments.

**Figure 10:**
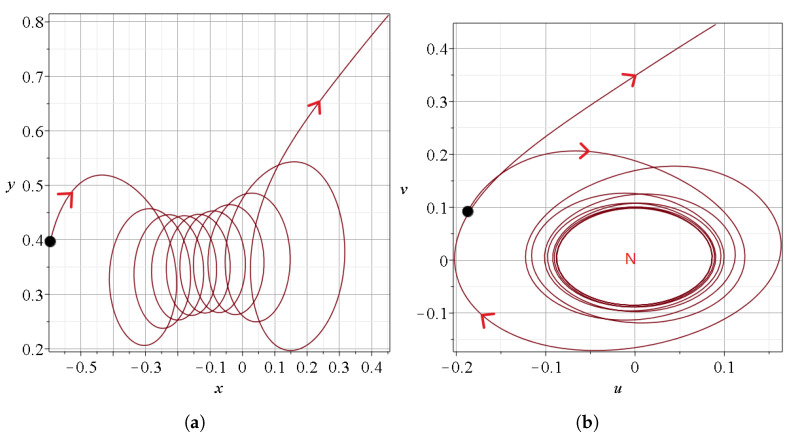
A zoom in of the Bohmian vortex (**a**) in the inertial frame of reference and (**b**) in the frame of reference of the moving nodal point (k=1), which is the center of the vortex.

The form of the spiral sections of the trajectories are shown in Figure 10a in the inertial frame of reference (x,y) and Figure 10b in the frame of reference of the moving nodal point k=1. We see that the moving particle forms loops around the nodal point k=1, and later it goes away. The trajectories during the approaches to N,X and *Y* without a vortex are similar to those of Figure 1a–c.

We conclude that every ’event’ in the motion of a particle consists of a number of approaches to a number of nearby nodal points and the corresponding *X* and *Y* points during a relatively short interval of time. During that time, the trajectory may form a loop or a spiral, but it may only show some irregularity (Figure 4a and Figure 8a). The sequence of the $k^{\prime }s$ of nearby approaches is not the same during different events. If the approaches are close, then they produce variations in the stretching number *a* and introduce chaos. However, distant approaches produce very small deviations in the trajectory.

### 2.3. Chaos and Ergodicity

In our previous works [25,50], we studied in detail the long-time behavior of the chaotic trajectories. We found that, for a given non-zero entanglement, all chaotic trajectories have practically the same long-time point distribution. Specifically, if we consider a sufficiently large grid of square cells covering the support of the wavefunction and count how many times each trajectory passes through them, we observe a common pattern across all chaotic trajectories. Therefore, the Bohmian chaotic trajectories are ergodic, since a single trajectory is sufficient to characterize the long-time chaotic behavior of the system.

**Figure 11:**
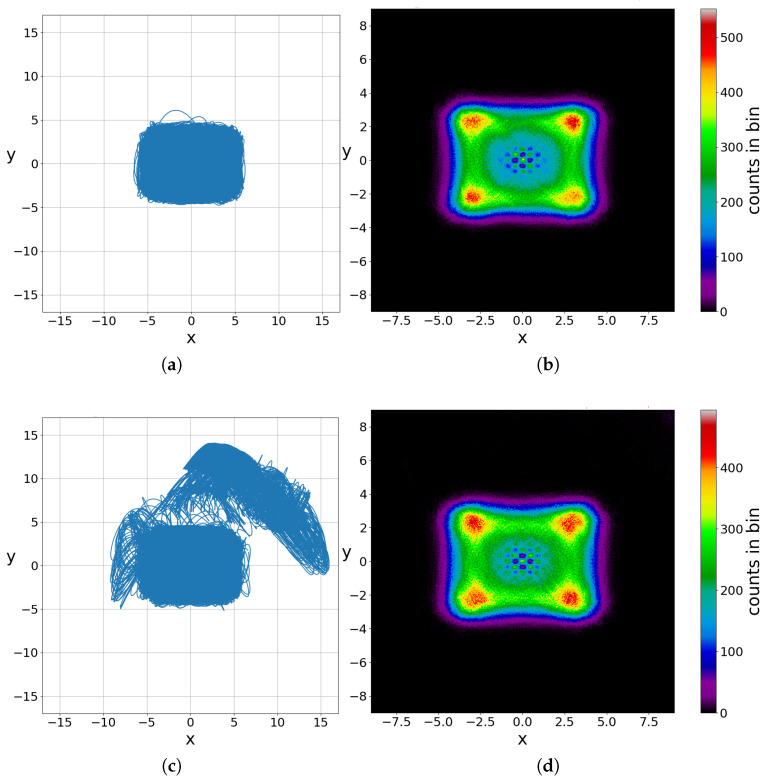
Two different chaotic trajectories in the maximally entangled state for $\omega_{x}=1,\omega_{y}=\sqrt{3}$ (**left panel**): In the first case, we start inside the effective support of the wavefunction (x(0)=y(0)=2), while in the second case, we start outside (x(0)=8,y(0)=10), in a region where the probability of finding a particle is negligible. Once the trajectory enters the central region, it remains there. The probability for the inverse process is extremely small. Their long limit colorplots (**right panel**) are practically the same at $t=3\times 10^{5}$, i.e., they are ergodic.

We note, however, that in classical dynamical systems, ergodicity is defined within a bounded phase space. In the quantum case, the phase space is in principle unbounded, as the support of the wavefunction extends to infinity. Nonetheless, for all practical purposes, the support is effectively bounded within a finite region around the origin. For example, working in the square region [−6,6]×[−6,6], we find that ${\left|\Psi \right|}^{2}\geq 10^{-21}$, ensuring that all significant dynamics of the system are taken into account. Outside this region, the probability of finding a particle is effectively zero. Conversely, if we consider a chaotic trajectory that starts far from the origin and integrate it for a sufficiently long time, we observe that it eventually enters the central region and remains there practically indefinitely. In Figure 11a,c, we present two trajectories corresponding to two different initial conditions, one inside the central region and one outside, that lead to chaotic trajectories. Then, we calculate their long-time $\left(t=3\times 10^{5}\right)$ colorplots (Figure 11b,d). The colorplots represent the number of points of a trajectory at successive steps Δt=0.05 in a dense grid of squares with side length equal to 0.05. The darker the color of a bin in our grid, the smaller the number of points of the trajectory at this bin. We see that although they correspond to different initial conditions, they have acquired practically the same form for this given time and with a very small difference in the counts inside the bins of the grid, i.e., they are ergodic.

## 3. Ordered Trajectories

**Figure 12:**
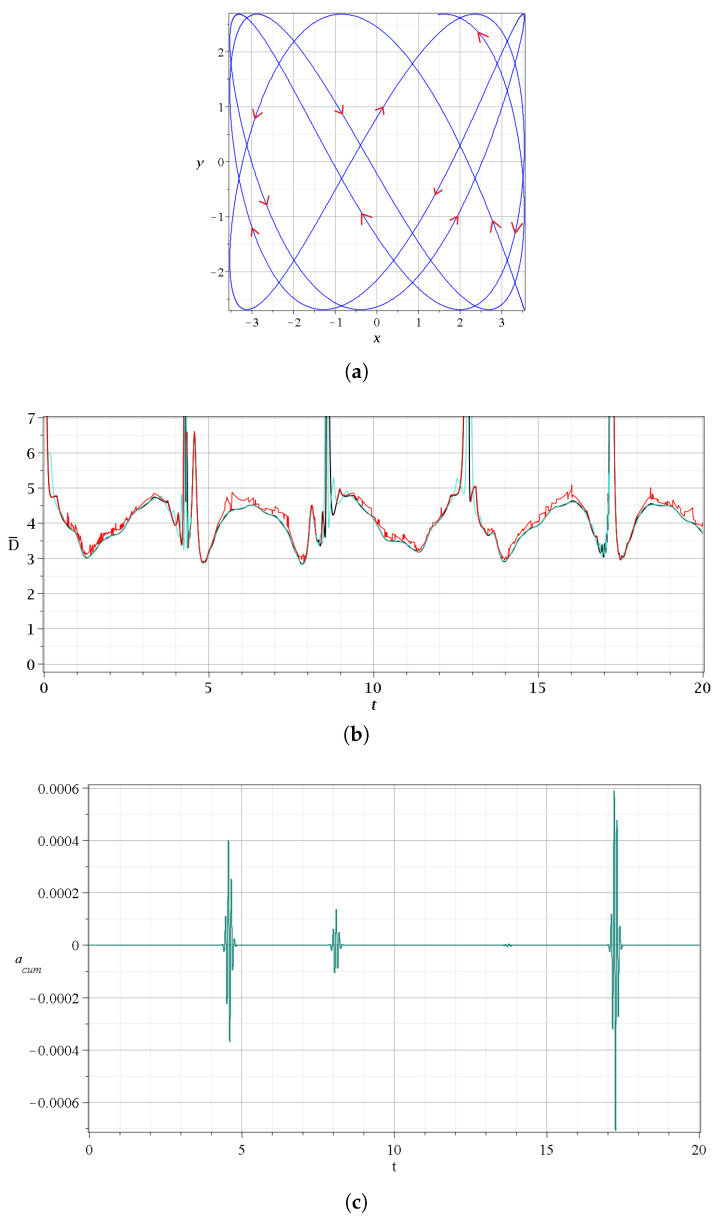
(**a**) An ordered trajectory in the case with $c_{2}=0.001,\omega_{x}=1,\omega_{y}=\sqrt{3}$, with initial conditions x(0)=3.54,y(0)=−2.69 for a time interval t=[0,20], following the directions of the red arrows. (**b**) The corresponding distances from the nearest point *N* (black), *X* (red) and *Y* (green). (**c**) The corresponding changes in the cumulative stretching number $a_{cum}=\sum a$, which are very small, of order $5\times 10^{-4}$ or smaller.

Ordered trajectories appear when the entanglement is less than its maximum value ($c_{2}=\sqrt{2}/2$). These trajectories are slightly deformed Lissajous figures (Figure 12a). In such a case, the approaches to the critical points N,X,Y are very distant (we see in Figure 12b that $\bar{D}>3$), and their effect is very small. The values of *a* at these approaches are very small and of order 0.0005 or smaller.

In Figure 12c, we give the $a_{cum}$ at every event. We see that after every event the $a_{cum}$ goes again close to zero. Therefore, the values of $\chi =\frac{1}{t}\sum a$ decrease further as time increases and tend to LCN=0 as *t* tends to infinity. In fact, χ decreases approximately as $∼t^{-1}$ (plotted on a double logarithmic scale).

A different class of ordered trajectories is found if the ratio $\omega_{y}/\omega_{x}$ is rational. If $\omega_{x}=s_{1}\omega$ and $\omega_{y}=s_{2}\omega$ the trajectory has a period T=2π/ω. During the time T/2, the trajectory undergoes a number of events (approaches to the N,X,Y points), and the stretching number *a* has some variations during every event. However, at t=T/2, the trajectory stops (i.e., dx/dt=dy/dt=0), and then it retraces its way along the same path, undergoing the same events with the opposite values of *a* until it reaches its origin. Therefore, the total sum of the values of *a* is zero, and consequently LCN=0. In Appendix B, we give a proof of the fact that the value of χ→0 when $\omega_{x}/\omega_{y}$ is a rational number, while it does not go to zero when this ratio is an irrational number.

## 4. Entanglement vs. Chaos

In [50], we conducted an extensive investigation of chaos and order in entangled qubits. More specifically, we focused on the influence of chaotic and regular dynamics on the establishment of the Born rule when the initial distribution of Bohmian particles deviates from it [51,52,53,54,55]. To this end, we distinguished between regular and chaotic trajectories within the Born distribution for each given value of entanglement.

Our results show that as the degree of entanglement increases, the proportion of chaotic (and ergodic) trajectories also increases, reaching 100% in the case of maximal entanglement. Consequently, in this regime, any arbitrary initial distribution of particles will evolve toward the Born distribution after a long time. However, in the case of a partially entangled state, there always exists a fraction of regular trajectories. These must be taken into account, both in terms of their proportion and of their specific locations on the Born distribution, if one aims to recover the Born rule from an arbitrary initial particle distribution.

In the present work, we take a step further by investigating how entanglement affects the degree of chaos exhibited by the chaotic trajectories themselves. Specifically, we compute the LCN of a large number of chaotic trajectories across a range of entanglement values, aiming to determine the impact of entanglement on the degree of chaos. This task is well known for its numerical difficulty, due to the accumulation of round-off errors during long-time integrations, as well as the considerable computational time required to obtain reliable estimates of the LCN. In the present work all relevant numerical computations were performed using an implementation of the (explicit) Dormand-Prince method. The absolute tolerance used was at most $a_{\mathrm{tol}}=10^{-9}$, although the minimum integration step size was always kept higher than $h=10^{-9}$ because some orbits could prove to be extremely stiff, and would be impossible to integrate without providing a lower bound for the step size. The stretching number was calculated by re-normalizing the variational equations every Δt=0.05.

**Figure 13:**
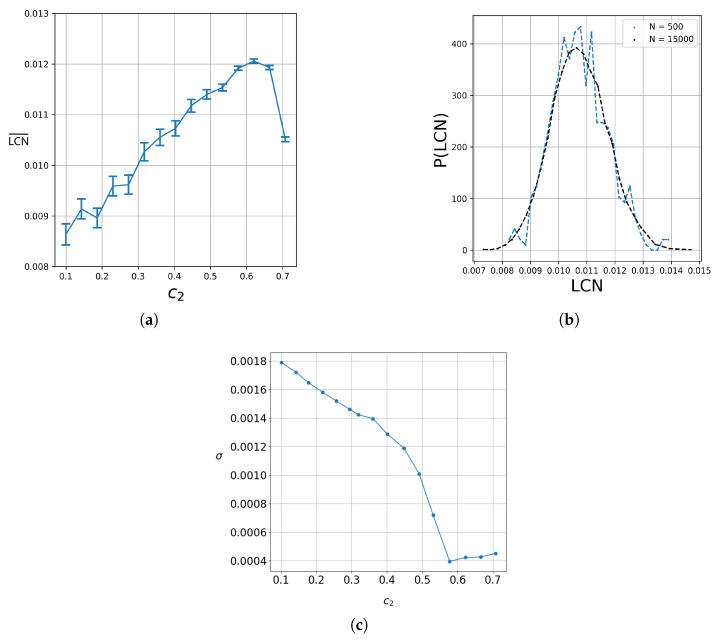
(**a**) The mean LCN for 400 trajectories up to t=100,000 for $\omega_{x}=1,\omega_{y}=\sqrt{3}$ and the corresponding error bars for various values of the entanglement. (**b**) The probability density P(LCN) in the case of maximum entanglement for 500 (blue), and 15,000 (black) trajectories uniformly sampled in the grid [−4,4]×[−4,4] for t=100,000. (**c**) The standard deviations of the LCN distributions in samples of 400 trajectories as a function of the entanglement for t=100,000.

It should be noted that in a previous study [20], a related analysis suggested no clear relationship between the degree of entanglement and chaos. We verified this in our present study. Indeed, as we show in Figure 13a, the mean LCN of chaotic trajectories in a set of 400 uniformly sampled trajectories in a square region x,y=−4.4 centered at the origin (x=0,y=0) does not exhibit monotonous dependence on quantum entanglement for all the values of the latter. In fact, between $c_{2}≃0.63$ and $c_{2}=0.66$, there is a maximum after which the mean LCN decreases as the entanglement tends to its maximum value ($c_{2}=\sqrt{2}/2$).

Furthermore, as we have shown in [24] (see also Appendix B), in the case of commensurable frequencies, all the trajectories of this system are periodic regardless of the degree of entanglement. Thus, in order to observe chaos, we have to work with non-commensurable frequencies. But, between two non-commensurable ratios $\omega_{x}/\omega_{y}$, there are infinitely many commensurable ratios which lead to LCN=0 for every trajectory in the phase space and regardless of the degree of entanglement, i.e., the value of LCN has infinitely many increases and decreases as $\omega_{x}/\omega_{y}$ changes. Consequently, there is no simple relation between the entanglement and the mean value of the LCN.

However, we found some interesting results concerning the distribution of the values of the LCN.

A notable result of our calculations is that the probability density given by P(LCN)=p(LCN)/d(LCN) (where p(LCN) is the probability of finding a given value of LCN in a small interval between LCN and LCN+d(LCN) (therefore ∫P(LCN)d(LCN)=1)) for a given entanglement of a large number of trajectories seems to have a Gaussian form. This can be seen in Figure 13b, which refers to 500 (blue) and 15000 (black) trajectories for $c_{2}=\sqrt{2}/2$ (maximum entanglement). We observe that as the number *N* of the trajectories increases, their distribution comes closer to a Gaussian. This Gaussian represents the trajectories along the error bar around the mean value of the LCN of every entanglement. This behavior can be understood in terms of the Central Limit Theorem (CLT) [56]. In chaotic systems, the LCN is computed as a time average along each trajectory. Due to the sensitivity to initial conditions and the mixing properties of chaos, each trajectory effectively samples many weakly correlated regions of phase space. As a result, the finite-time LCN behaves like the average of many weakly dependent random variables, which tends toward a normal distribution according to the CLT.

In Figure 13c, we show the standard deviation σ as a function of $c_{2}$. We see that σ decreases as the entanglement increases to $c_{2}=0.59$, and for larger $c_{2}$, it is almost constant. We verified that this happens in many independent repetitions of the experiment with 400 trajectories. This suggests that there is likely a relationship between entanglement and the LCN convergence time.

**Figure 14:**
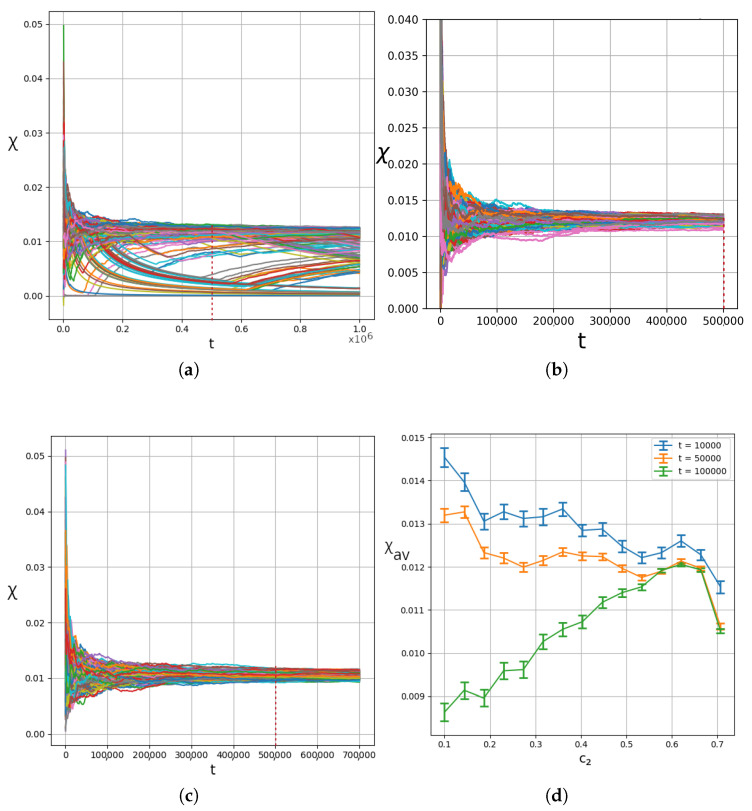
The finite time LCN of 400 trajectories uniformly sampled in the grid [−4,4]×[−4,4] for (**a**) $c_{2}=0.5$, (**b**) $c_{2}=0.6$, and (**c**) $c_{2}=\sqrt{2}/2$. (**d**) Average χ vs. entanglement for three different integration times, $t=10^{4}$ (blue), $t=5\times 10^{4}$ (orange) and $t=10^{5}$ (green). In all cases, $\omega_{x}=1,\omega_{y}=\sqrt{3}$. The thick dashed vertical lines indicate the time $t=5\times 10^{5}$.

In fact, in Figure 14a–c, we have the values of the finite time LCN of 400 trajectories for entanglements of 0.5,0.6 and 0.707. We clearly see that even after one million time units in the case of Figure 14a, the finite time LCN, χ has not reached its final value. On the other hand, in Figure 14b,c, where the entanglement is large and then maximized, the various trajectories have dispersions Δχ of their finite time LCN, which decrease in time. Based on these observations, we calculated Figure 14d, where we show the mean values and the error bars for the LCN at different entanglement levels, and for three different observation times: 10,000, 50,000, and 100,000 time units. There, we can clearly see that for small entanglements, we have large standard deviations around the mean value, as well as large differences in the mean values across the various observation times. However, as the entanglement increases, both the distance between the mean values and the size of the error bars decrease.

**Figure 15:**
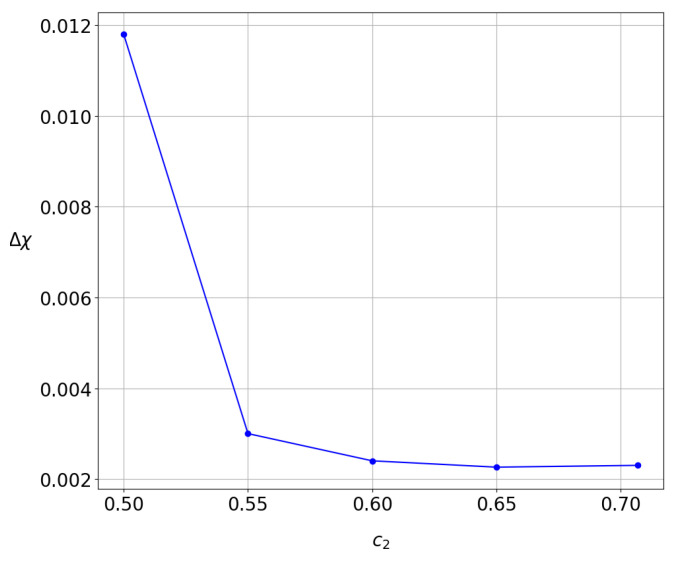
The range Δχ of the average χ at $t=5\times 10^{5}$ for various degrees of strong entanglement. The larger the entanglement, the smaller the Δχ.

As a consequence, the time needed for the convergence of χ to a value close to the final LCN decreases with the increase in the entanglement. This can be seen in Figure 15, where we give the range Δχ of the distribution of the values of χ at a fixed time $t=5\times 10^{5}$ as a function of $c_{2}$. We see that Δχ is large when $c_{2}=0.5,$ but it decreases for larger entanglements. Therefore, for relatively large entanglements, the convergence of the values of χ is faster. This result is significant and fully consistent with our previous findings for the evolution of the probability density of this system [25,50].

## 5. Conclusions

In the present paper, we studied the combined effect of the two kinds of unstable points in the Bohmian flow, the X-points (in the frame of reference of the moving node) and the Y-points (in the inertial frame of reference), in a system of two entangled qubits. The nodal points are infinitely many. They are located along a straight line and move in time, going to infinity when $\omega_{xy}t=K\pi$ (K∈Z) and coming back from the opposite side of infinity. Our new findings in the present paper are as follows:

1. Every nodal point is followed by two X-points (not only one), and between two neighboring nodal points, there is a Y-point.
2. The position of the Y-points has been given analytically, while the X-points were found only numerically.
3. Chaos is introduced at successive points, when a particle in a relatively small interval of time approaches successively a number of nodal points and their X-points, and also the Y-points between them. A set of such approaches occurs between every two approaches to infinity. Such a set of approaches to the N,X,Y is called an ‘event’. We studied some characteristic events in detail.
4. In most cases, when the particle approaches a nodal point *N*, it also approaches its X-point, which is then quite close to *N*. After that time, the particle goes further away from these points (*N* and *X*) and approaches a Y-point between this and a neighboring nodal point. After that, the particle starts to approach the neighboring nodal and X-point. This process is repeated with approaches to a number of *N* and *X* points and the *Y* points between them. The event of approaches terminates when the particle goes far from all the *N* points.
5. In some cases, when the particle is trapped in a very close region to the nodal point it follows a spiral motion (Bohmian vortex) around it. In this case, there is a significant production of chaos.
6. We provided some examples of the trajectories that lead to approaches to the points N,X,Y, both in the inertial frame (*x*, *y*) and in the frame (*u*, *v*) around a nodal point. The points *X* and *Y* of the same event produce one set of variations of the stretching number, and both contribute significantly to the production of chaos. But in general, the X-points are located at higher levels of the quantum potential surface than the Y-points, and the total force acting on the particle at *X* is larger than that at *Y*.
7. We gave an example of an ordered trajectory that remains far from the critical points N,X,Y. In this case, the stretching numbers are very small, and the LCN is zero when $\omega_{x}/\omega_{y}$ is rational. The moving particle may approach the points N,X,Y (not so close, however, as in the case of non-commensurable frequencies), but its trajectory is periodic.
8. We found that all the chaotic trajectories are ergodic, even when they start far from the central region in the x−y plane. Thus, while the general form of the colorplot in a chaotic trajectory is formed in a relatively short time (we observe that it covers the full available space of the effective support of the wavefunction), the value of the LCN itself needs a significantly large time to saturate on a positive number.
9. We found numerically that entanglement does not affect the value of the LCN in a simple and unique way. However, our simulations provided evidence that the increase in entanglement decreases the convergence time of the chaotic trajectories to the final positive value of the LCN.

The above results shed new light on the mechanisms responsible for chaos in Bohmian trajectories, highlighting the role of both X- and Y-type unstable points in the dynamics of entangled qubit systems. By analyzing how chaos arises through repeated approaches to these critical points, this study enhances our understanding of the structure and behavior of Bohmian flows. Moreover, it offers useful insight into the interplay between entanglement and chaos within the Bohmian framework, supporting our previous research on the influence of entanglement on the generation and structure of Bohmian chaos.

In our future research plans, we aim to explore the relationship between Bohmian chaos and the broader phenomena of quantum chaos, including signatures such as entanglement entropy growth [57], statistical properties of energy level spacings, and spectral correlations. Understanding this relationship is fundamental for developing a deeper and more unified view of quantum dynamical behavior [58,59].

## Figures and Tables

**Figure A1:**
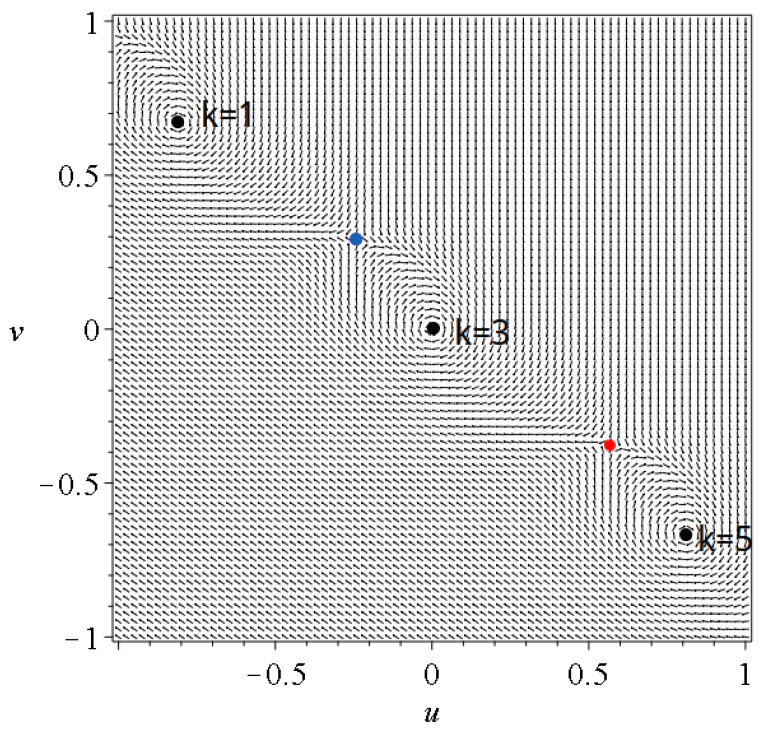
The Bohmian velocity field in the frame of reference of the nodal point k=3 for $c_{2}=\sqrt{2}/2$ at t=5.8. All the nodal points are shown with black dots. The X-point on the right of the node k=3 is marked red, while that on the left is marked blue.

## Data Availability

The datasets generated during the current study are available from the corresponding author upon reasonable request.

## Appendix A. Detection of the X-Points

The position of the X-points depends both on time and on the specific nodal point considered. To find their location, we first fix a time *t* and then transform to the reference frame of a chosen nodal point *N*. In this frame, we typically find that each nodal point is accompanied by two X-points in its (u,v) plane, located on each side of *N* (Figure A1). When we shift to the frame of the next or previous nodal point, we again observe an X-point on each side. Therefore, between any two successive nodal points, there are generally two X-points: one ahead of the first nodal point and one behind the second. When the velocities of the nodal points are small, the associated X-points are very close to each other. As the nodal velocities increase, the X-points tend to move closer to the nodal points themselves.

To understand the scattering events, we integrate the trajectory up to a fixed time using a constant time step. For the same time and step size, we store the trajectories of a large number of nodal points (i.e., for many values of *k*). At each time step, we identify the nodal point closest to the particle trajectory. We then switch to its (u,v) plane, compute the corresponding X-points, determine which of them is nearest to the trajectory, and store its distance from the trajectory.

The implementation of the above algorithm becomes very difficult from time to time when the nodal points acquire very large velocities before escaping to infinity. There we have NPXPCs with extremely small distance between *N* and *X*, and the numerical convergence to the position of the X-point is slow, if not impossible, if we do not provide the computer with a highly accurate guess solution.

## Appendix B. The Case of Ordered Trajectories

The Bohmian equations of motion in the case of two qubits are

(A1) $$\begin{array}{c} \frac{dx}{dt}=-\frac{\sqrt{2\omega_{x}}}{G}\alpha_{0}\left[A\cos\left(\omega_{x}t\right)+B\sin\left(\omega_{x}t\right)\right], \end{array}$$

(A2) $$\begin{array}{c} \frac{dy}{dt}=\frac{\sqrt{2\omega_{y}}}{G}\alpha_{0}\left[A\cos\left(\omega_{y}t\right)+B\sin\left(\omega_{y}t\right)\right], \end{array}$$

where

(A3) $$\begin{array}{c} A=2c_{1}c_{2}e^{2f_{x}+2f_{y}}\sin\left(2(g_{x}-g_{y})\right),\\ B=c_{1}^{2}e^{4f_{x}}-c_{2}^{2}e^{4f_{y}},\\ G=2c_{1}c_{2}e^{2f_{x}+2f_{y}}\cos\left(2(g_{x}-g_{y})\right)+e^{4f_{y}}{c_{2}}^{2}+e^{4f_{x}}{c_{1}}^{2} \end{array}$$

with

(A4) $$\begin{array}{c} f_{x}=\sqrt{2\omega_{x}}a_{0}\cos\left(\omega_{x}\;t\right)x,\;f_{y}=\sqrt{2\omega_{y}}a_{0}\cos\left(\omega_{y}\;t\right)y,\\ g_{x}=\sqrt{2\omega_{x}}a_{0}\;\sin\left(\omega_{x}\;t\right)x,\;g_{y}=\sqrt{2\omega_{y}}a_{0}\;\sin\left(\omega_{y}\;t\right)y \end{array}$$

and $c_{1}^{2}+c_{2}^{2}=1$. We assumed $a_{0}=5/2$, $\omega_{x}=1$, $\omega_{y}=\sqrt{3}$.

When t=0, we have $g_{1}=g_{2}=0$, therefore A=0 and $\dot{x}=\dot{y}=0$. For $t^{\prime }=-t$, we have the same $f_{1}$, $f_{2}$ but opposite $g_{1}$ and $g_{2}$. Then, we find an opposite *A* and opposite $\dot{x}$, $\dot{y}$. Therefore, we describe the same trajectory in the opposite direction.

If $\omega_{x}/\omega_{y}$ is irrational, there is no point in the trajectory where both $\dot{x}$ and $\dot{y}$ are zero. However, if the fraction is rational, we have $\dot{x}=\dot{y}=0$ not only for t=0 but also for every kT/2=k2π/ω,k=1,2,…. In fact, at t=T/2, the moving particle stops and then retraces the same trajectory backwards up to t=T.

Then, the values of α have the same absolute values but with an opposite sign. This guarantees that the LCN after one period is zero. Beyond the first period, the values of α are the same as in the first period, but in calculating χ, the denominator *t* is now t+T, therefore the value of χ is smaller. The value of χ is zero at all the multiples of t=T, but the amplitude of χ between any two zero values becomes smaller and tends to zero as t→∞.

## References

[1] De Broglie L. (1926). Sur la possibilité de relier les phénomenes d’interférence et de diffractiona la théorie des quanta de lumiere. C. R. Acad. Sci..

[2] Bohm D. (1952). A Suggested Interpretation of the Quantum Theory in Terms of “Hidden” Variables. I. Phys. Rev..

[3] Bohm D. (1952). A Suggested Interpretation of the Quantum Theory in Terms of “Hidden” Variables. II. Phys. Rev..

[4] Holland P.R. (1995). The Quantum Theory of Motion: An Account of the de Broglie-Bohm Causal Interpretation of Quantum Mechanics.

[5] Bohm D., Hiley B.J. (2006). The Undivided Universe: An Ontological Interpretation of Quantum Theory.

[6] Dürr D., Goldstein S., Tumulka R., Zanghi N. (2004). Bohmian mechanics and quantum field theory. Phys. Rev. Lett..

[7] Kocsis S., Braverman B., Ravets S., Stevens M.J., Mirin R.P., Shalm L.K., Steinberg A.M. (2011). Observing the average trajectories of single photons in a two-slit interferometer. Science.

[8] Pladevall X.O., Mompart J. (2012). Applied Bohmian Mechanics: From Nanoscale Systems to Cosmology.

[9] Haake F. (2010). Quantum Signatures of Chaos.

[10] Wimberger S. (2014). Nonlinear Dynamics and Quantum Chaos.

[11] Robnik M. (2016). Fundamental concepts of quantum chaos. Eur. Phys. J. Spec. Top.

[12] Bohigas O., Giannoni M.J., Schmit C. (1984). Characterization of chaotic quantum spectra and universality of level fluctuation laws. Phys. Rev. Lett..

[13] Hashimoto K., Murata K., Yoshii R. (2017). Out-of-time-order correlators in quantum mechanics. J. High Energy Phys..

[14] Iacomelli G., Pettini M. (1996). Regular and chaotic quantum motions. Phys. Lett. A.

[15] Frisk H. (1997). Properties of the trajectories in Bohmian mechanics. Phys. Lett. A.

[16] Falsaperla P., Fonte G. (2003). On the motion of a single particle near a nodal line in the de Broglie–Bohm interpretation of quantum mechanics. Phys. Lett. A.

[17] Wisniacki D.A., Pujals E.R. (2005). Motion of vortices implies chaos in Bohmian mechanics. Europhys. Lett..

[18] Wisniacki D., Pujals E., Borondo F. (2007). Vortex dynamics and their interactions in quantum trajectories. J. Phys. A.

[19] Borondo F., Luque A., Villanueva J., Wisniacki D.A. (2009). A dynamical systems approach to Bohmian trajectories in a 2D harmonic oscillator. J. Phys. A.

[20] Cesa A., Martin J., Struyve W. (2016). Chaotic Bohmian trajectories for stationary states. J. Phys. A.

[21] Efthymiopoulos C., Kalapotharakos C., Contopoulos G. (2009). Origin of chaos near critical points of quantum flow. Phys. Rev. E.

[22] Makowski A.J., Frackowiak M. (2001). The simplest non-trivial model of chaotic causal dynamics. Acta Phys. Pol. B.

[23] Tzemos A.C., Contopoulos G. (2022). Bohmian chaos in multinodal bound states. Found. Phys..

[24] Tzemos A.C., Contopoulos G., Efthymiopoulos C. (2019). Bohmian trajectories in an entangled two-qubit system. Phys. Scr..

[25] Tzemos A.C., Contopoulos G. (2020). Ergodicity and Born’s rule in an entangled two-qubit Bohmian system. Phys. Rev. E.

[26] Nielsen M.A., Chuang I.L. (2004). Quantum Computation and Quantum Information.

[27] Tzemos A.C., Contopoulos G. (2020). Chaos and ergodicity in an entangled two-qubit Bohmian system. Phys. Scr..

[28] Strang G. (1993). Introduction to Linear Algebra.

[29] Coudène Y. (2016). Ergodic Theory and Dynamical Systems.

[30] Avanzini F., Moro G.J. (2017). Quantum Molecular Trajectory and Its Statistical Properties. J. Phys. Chem. A.

[31] Tzemos A.C., Contopoulos G. (2023). Unstable points, ergodicity and Born’s rule in 2D Bohmian systems. Entropy.

[32] Horodecki R., Horodecki P., Horodecki M., Horodecki K. (2009). Quantum entanglement. Rev. Mod. Phys..

[33] Erhard M., Krenn M., Zeilinger A. (2020). Advances in high-dimensional quantum entanglement. Nat. Rev. Phys..

[34] Benatti F., Floreanini R., Franchini F., Marzolino U. (2020). Entanglement in indistinguishable particle systems. Phys. Rep..

[35] Chang K.C., Sarihan M.C., Cheng X., Zhang Z., Wong C.W. (2023). Large-alphabet time-bin quantum key distribution and Einstein–Podolsky–Rosen steering via dispersive optics. Quantum Sci. Technol..

[36] Chang K.C., Cheng X., Sarihan M.C., Wong C.W. (2025). Recent advances in high-dimensional quantum frequency combs. Newton.

[37] Braverman B., Simon C. (2013). Proposal to observe the nonlocality of Bohmian trajectories with entangled photons. Phys. Rev. Lett..

[38] Xiao Y., Kedem Y., Xu J.S., Li C.F., Guo G.C. (2017). Experimental nonlocal steering of Bohmian trajectories. Opt. Express.

[39] Zander C., Plastino A. (2018). Revisiting Entanglement within the Bohmian Approach to Quantum Mechanics. Entropy.

[40] Foo J., Lund A.P., Ralph T.C. (2024). Measurement-based Lorentz-covariant Bohmian trajectories of interacting photons. Phys. Rev. A.

[41] Parmenter R.H., Valentine R. (1995). Deterministic chaos and the causal interpretation of quantum mechanics. Phys. Lett. A.

[42] Garrison J., Chiao R. (2008). Quantum Optics.

[43] Ballentine L.E. (2014). Quantum Mechanics: A Modern Development.

[44] Ghose P., Majumdar A., Guha S., Sau J. (2001). Bohmian trajectories for photons. Phys. Lett. A.

[45] Tzemos A., Contopoulos G. (2022). Bohmian quantum potential and chaos. Chaos Solitons Fractals.

[46] Bohm D., Hiley B.J. (1984). Measurement understood through the quantum potential approach. Found. Phys..

[47] Goldstein S., Struyve W. (2014). On quantum potential dynamics. J. Phys. A.

[48] Dennis G., de Gosson M.A., Hiley B.J. (2015). Bohm’s quantum potential as an internal energy. Phys. Lett. A.

[49] Hojman S.A., Asenjo F.A., Moya-Cessa H.M., Soto-Eguibar F. (2021). Bohm potential is real and its effects are measurable. Optik.

[50] Tzemos A., Contopoulos G. (2021). The role of chaotic and ordered trajectories in establishing Born’s rule. Phys. Scr..

[51] Valentini A. (1991). Signal-locality, uncertainty, and the subquantum H-theorem. I. Phys. Lett. A.

[52] Valentini A. (1991). Signal-locality, uncertainty, and the subquantum H-theorem. II. Phys. Lett. A.

[53] Valentini A., Westman H. (2005). Dynamical origin of quantum probabilities. Proc. Roy. Soc. A.

[54] Dürr D., Struyve W. (2020). Do Wave Functions Jump? Perspectives of the Work of GianCarlo Ghirardi.

[55] Lustosa F.B., Pinto-Neto N., Valentini A. (2023). Evolution of quantum non-equilibrium for coupled harmonic oscillators. Proc. R. Soc. A.

[56] Johnson R.A., Miller I., Freund J.E. (2011). Probability and Statistics for Engineers.

[57] Vidmar L., Rigol M. (2017). Entanglement entropy of eigenstates of quantum chaotic Hamiltonians. Phys. Rev. Lett..

[58] Wang X., Ghose S., Sanders B.C., Hu B. (2004). Entanglement as a signature of quantum chaos. Phys. Rev. E.

[59] Benenti G. (2009). Entanglement, randomness and chaos. Riv. Nuovo Cim..

